# Existing and Novel Biomaterials for Bone Tissue Engineering

**DOI:** 10.3390/ijms24010529

**Published:** 2022-12-28

**Authors:** Paweł Dec, Andrzej Modrzejewski, Andrzej Pawlik

**Affiliations:** 1 Department of Plastic and Reconstructive Surgery, 109 Military Hospital, 71-422 Szczecin, Poland; 2 Department of Surgery, Pomeranian Medical University, 71-422 Szczecin, Poland; 3 Department of Physiology, Pomeranian Medical University, 70-111 Szczecin, Poland

**Keywords:** biomaterials, bone tissue, engineering

## Abstract

The treatment of bone defects remains one of the major challenges in modern clinical practice. Nowadays, with the increased incidence of bone disease in an aging population, the demand for materials to repair bone defects continues to grow. Recent advances in the development of biomaterials offer new possibilities for exploring modern bone tissue engineering strategies. Both natural and synthetic biomaterials have been used for tissue repair. A variety of porous structures that promote cell adhesion, differentiation, and proliferation enable better implant integration with increasingly better physical properties. The selection of a suitable biomaterial on which the patient’s new tissue will grow is one of the key issues when designing a modern tissue scaffold and planning the entire treatment process. The purpose of this article is to present a comprehensive literature review of existing and novel biomaterials used in the surgical treatment of bone tissue defects. The materials described are divided into three groups—organic, inorganic, and synthetic polymers—taking into account current trends. This review highlights different types of existing and novel natural and synthetic materials used in bone tissue engineering and their advantages and disadvantages for bone defects regeneration.

## 1. Introduction

Bone tissue accounts for about 15% of the total body weight. The structure of bone tissue is two-layered: the outer layer is cortical bone, which is characterized by high mechanical resistance and the inner layer is spongy bone, which has a high porosity coefficient of about 80–90% [1]. Bone hardness is provided by the extracellular collagen matrix, which is saturated with inorganic calcium phosphate molecules, mainly hydroxyapatite Ca_10_(PO_4_)_6_(OH)_2_. Calcium and phosphate make up about 65–70% of bone dry weight. Bone tissue has a Ca/P ratio of 1.67, but this can vary from 1.37 to 1.87 due to the presence of additional mineral ions [2].

Bone tissue is an extremely metabolically active connective tissue capable of continuous resorption and remodeling, but the regeneration process often requires additional support for large bone defects, such as trauma, tumour excision, and age-related diseases. Small bone defects usually heal without additional intervention [3]. However, when the bone defect exceeds a critical size threshold (approximately >2 cm) or more than 50% loss of bone circumference [4], it will result in a lack of fusion, abnormal fusion, or pathological fracture [5]. Tissue engineering is an interdisciplinary field that combines the knowledge of cells, engineered materials, and biochemical factors to develop a suitable artificial cellular scaffold to maintain or regenerate damaged tissues [6,7].

**Figure 1:**
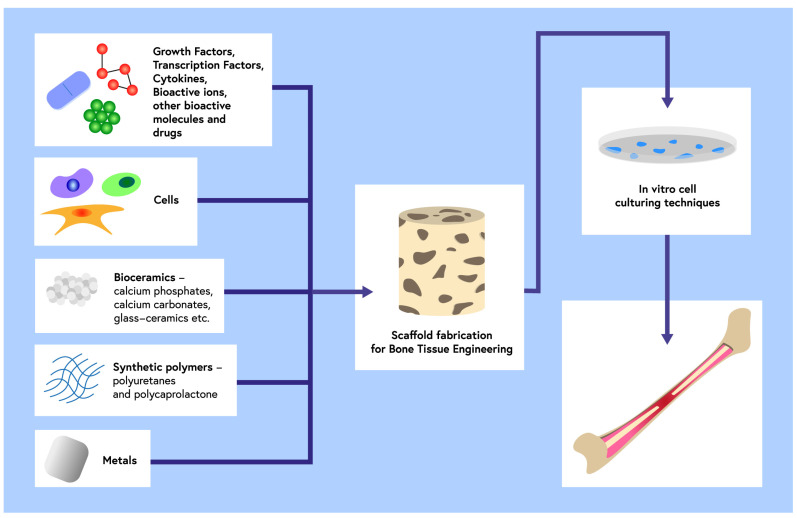
The strategies of bone tissue engineering.

Biomaterials are a broad group of materials with different compositions, structures, and physical properties, distinguished by good tolerance by the human body and some of which have the ability to permanently fuse with tissue or promote its regeneration (such as hydroxyapatite ceramics, bio-glasses, or modified carbon materials) (Figure 1).

Tissue can respond to implants with different types of local reactions. Among the most common are absorption, excretion, and otorrhea. The course and effectiveness of implant penetration depends on the structure and shape of the implant. Knitted, spongy, and porous implants indent by overgrowing with ingrown connective tissue, regardless of whether they are non-resorbable or resorbable. Solid implants indent by being completely or partially obliterated by connective tissue. There may be gelling cells in the immediate vicinity of the implant; however, their number depends on the irritant effect of the implant and the presence of particles of the implanted material. This also applies to the thickness of the created connective tissue capsule around the implant. The more inert the implant is in terms of its physicochemical properties, the lower the local reaction of the body and the more likely the process of implantation will proceed with almost no inflammatory reaction. It is characterised by a short and low intensity exudative phase, which quickly turns into a proliferative phase, leading to the formation of a thin, fibrous connective tissue capsule. It can therefore be concluded that the implant is characterised by a high degree of biocompatibility. A biomaterial should exhibit biological compatibility (biocompatibility). This means harmony of interactions within animate matter. A biomaterial with optimal biotolerance does not cause acute or chronic reactions or inflammation and does not interfere with the normal differentiation of the surrounding tissues. Most often, the concept of biotolerance is associated with the initiation of toxicological and immunological reactions and tissue irritation effects. Biocompatibility is a determination of the severity of the local tissue response to an implant. Implant studies make it possible to trace the process of implant engraftment at the cellular level over a certain period of time (microscopic studies) and to trace any changes occurring in the implant itself (scanning microscope studies and physicochemical studies of the implant after its residence in tissues). An important feature of artificial biomaterials is their biodegradability.

A feature of a good bone scaffold should be degradation at a controlled rate, allowing new bone tissue to grow. In addition, the degradation products should be non-toxic [8]. In addition, bone scaffolds should have mechanical properties similar to human bone to ensure proper transmission of forces and loads [9,10].

To date, hydroxyapatite bioceramics have found the widest application in bone tissue substitution in the form of porous shapes and pellets. Porous synthetic hydroxyapatite binds strongly to bone, as a so-called biological bond can occur in addition to chemical bonding due to the ingrowth of living tissue into the pores of the implant. This allows stable attachment of the implant in the defect, thus protecting the implant from becoming loose. The requirement for living tissue to grow into the pores of the biomaterial and maintain its viability is a sufficiently large open pore size. It is assumed that the minimum size of open pores to allow the formation of a biological connection between the implant and the bone is 100 μm. If the pores reach 200 μm, then the development of osteons is possible within the bone implant. For good integration of implants into bone, it is important that there is a network of interconnected pores in the surface layer, without the presence of constrictions. With proper immobilisation, vascularised bone can be expected to grow into such pores. Commercially available porous hydroxyapatite implants have a flexural strength of 2–11 MPa, a compressive strength of 2–100 MPa, and a tensile strength of about 3 MPa. The mechanical parameters of the implanted hydroxyapatite biomaterial improve after overgrowth with natural infiltrating bone tissue. It has been shown that if the pores are 50–60% filled with cortical bone, the flexural strength increases to 40–60 MPa [11].

## 2. Inorganic Materials

Inorganic materials are divided into metallic and non-metallic materials, which have high mechanical strength and are not easily deformed or degraded; some require a secondary operation to remove them. Metals present the most favourable mechanical and strength properties among the previously discussed biomaterials. This makes them suitable for use in orthopaedics and prosthetics as components exposed to high compressive forces. It is essential for bone implants to have mechanical properties comparable to natural bone, as this can lead to atrophy of the natural tissue surrounding the implant. Metals exhibit higher mechanical properties (including stiffness and hardness) than the natural structure, so it is common for the implant to take over the transferred loads and reduce bone density.

Metallic materials are used for bone repair due to their favorable mechanical properties. Metal-based biomaterials include titanium, tantalum, cobalt, and magnesium alloys. Some of the most commonly used in clinical practice are Ti6Al4V titanium alloys [12]. Pure titanium has high corrosion resistance in physiological environments, but poor strength limits its clinical use. Compared to pure titanium, Ti6Al4V titanium alloy has optimal flexibility and mechanical resistance [13].

However, titanium is not corrosion-resistant and bonds to bone, meaning that the addition of surface coatings is often required to increase its bioactivity and corrosion resistance, including non-adhesive coatings and composite coatings [14]. Both magnesium-based alloys and zinc-based alloys are biodegradable materials used to treat bone injuries [15,16]. Magnesium is an essential element in the human body that is involved in cellular metabolism. Magnesium alloys exhibit favourable degradability, plasticity, and mechanical strength, thus avoiding secondary surgery after implantation. In an experimental study, a Mg^2+^ scaffold and a hydroxyapatite scaffold were implanted into rabbit femurs, respectively. The results showed that both scaffolds had good biocompatibility [17].

Bioceramics for the treatment of bone defects have been widely studied based on their favourable biocompatibility, biodegradability, osteoconductivity, and osteoinduction [18]. Calcium phosphate (CaP)-based ceramics are basic bone repair materials with excellent osteoinductive and osteoconductive properties; examples include hydroxyapatite (HAP), tricalcium β-phosphate (β-TCP), calcium polyphosphate (CPP), and biphasic calcium phosphate (BCP). Powdered bioceramic materials cannot be directly used to repair bone defects due to their rapid degradation and volume loss. Therefore, various porous, three-dimensional tissue engineering scaffolds have been prepared and proven to have sufficient mechanical support, nutrient exchange, and ingrowth tissue induction to enable the use of bioceramics in the treatment of large bone defects. A comparison of the various biomaterials used for implants shows that ceramic materials have been found to be relatively brittle materials with low flexural strength [19]. Ceramic materials are not resistant to dynamic loads and do not exhibit deformability. High hardness and good abrasion and corrosion resistance in tissue and body fluid environments minimise, but do not completely eliminate, wear and tear on ceramic biomaterials after long-term use.

The research conducted so far has shown that some metals used in biomaterials can have multidirectional effects.

Studies confirm strontium’s dual mechanism of action. Strontium both stimulates bone formation and inhibits bone resorption [20,21]. Previous studies have shown that strontium influences bone remodeling by regulating osteoblast and osteoclast function through the BMP-2/Smad1 and OPG/RANKL signaling pathways [22,23]. Sr is believed to have both osteogenic (anabolic) and anti-absorptive (catabolic) effects [24,25].

Many studies have shown that the addition of Sr can stimulate the differentiation of mesenchymal stem cells or other osteoblast lineages [26]. The expression of osteoblast markers (alkaline phosphatase [ALP], bone sialoprotein, and osteocalcin) has been increased to promote bone formation [27,28], while limiting osteoclast differentiation and proliferation [29]. Titanium-based alloys are commonly used for load-bearing applications (such as total joint replacement and fracture fixation components), but carry a risk of loosening, especially when implanted in osteoporotic bones. Tao et al. conducted an in vivo study on rats with osteoporosis caused by ovarian resection and found that the HAP coating containing strontium on shaped titanium implants was superior to the HAP coating without Sr in terms of new bone formation and resistance to physical forces [30]. Liu et al. produced biodegradable Mg–Cu alloys, which have been shown to promote bone formation (in vitro model of mouse cranial pre-osteoblasts) and angiogenesis (in vitro model of human umbilical vein endothelial cells) due to the sustained release of Cu^2+^ and Mg^2+^ and they have long-lasting antibacterial properties [31]. Sun and Chen modified titanium surfaces with zinc to produce biomaterials with great potential for orthopedic applications. They showed that the release of Zn ions from the titanium implant promoted osteogenic differentiation leading to a marked acceleration of bone formation [32,33]. Similarly, Thian et al. synthesised Zn-doped HA and demonstrated the enhanced proliferation and differentiation potential of human adipose tissue-derived mesenchymal stem cells (ADSCs) on biomaterials made from Zn-doped HA [34]. In contrast, Andersen et al. showed that an Sr-modified titanium implant surface improved the osteogenic differentiation of human DPSCs and osteointegration of the implant into the femur on a rat model. Fluoride ions have also shown the ability to promote osteogenic differentiation of osteoblastic cells through the Runx2/Osterix pathway and are therefore often used for HA substitution [35,36]. A new bone substitute consisting of a combination of CaP and Al_2_O_3_ has good compatibility both in vivo and in vitro. Aluminium oxide has an extremely low degradation rate compared to CaP; a sufficiently high concentration can lead to better stability and biomechanical resistance than pure CaP [37].

Bioceramic scaffolds coated with human bone morphogenetic protein-2 (BMP-2) promote the induction of osteoinduction and bone remodeling. A calcium silicate/calcium phosphate scaffold with macropores and micropores saturated with BMP-2 has been developed [38]. In this study, the authors found that the microporous scaffold preserved the secondary structure and biological activity of BMP-2 and the local release of BMP-2 promoted new bone formation and the material had many clinical successes. However, it is worth noting that serious side effects associated with ectopic or unwanted bone formation in some situations have led to the FDA being increasingly discouraged from approving the use of such materials [39]. Techniques using porous bioceramic scaffolds containing autologous mesenchymal stem cells appear promising [40]. Bioceramic microporous HAp scaffolds significantly increase mesenchymal cell adhesion and viability, alkaline phosphatase (ALP) activity, and mRNA expression levels of osteogenic markers and angiogenic factors. In vivo bone regeneration results in rat models of cranial defects confirmed that combining a porous surface with mesenchymal cells can significantly improve both osteogenesis and angiogenesis compared to a control HAp bioceramic scaffold with a traditional smooth surface [41]. Strontium (Sr), zinc (Zn), magnesium (Mg), and silicium (Si) are believed to be essential trace elements for bone growth and mineralisation. Admixtures of these ions used to make bioceramic scaffolds have the potential to stimulate the growth of bone marrow mesenchymal stem cells and, most importantly, they significantly increased ALP activity and osteogenesis-related gene expression compared to β-TCP-based bioceramics. The results suggest that the specific combination of bioactive ions (e.g., Sr, Zn, Si) in bioceramics is an effective way to improve the bioactivity of biomaterials and the form of materials and surface properties were factors influencing cellular response. The results of the studies showed that Sr- and Si-containing bioceramics could increase ALP activity and the expression of Col 1, OCN, Runx2, and angiogenic factors, including VEGF and Ang-1. Sr and Si ions showed synergistic effects on osteogenesis, osteoclastogenesis, and angiogenesis, which was attributed to the dominant effect of Sr ions on increasing angiogenesis and inhibiting osteoclastogenesis and the dominant effect of Si ions on stimulating osteogenesis. An in vivo study using critical defects in the mandible of rat models of OVX showed that Sr- and Si-containing bioceramics could significantly enhance bone formation and mineralisation compared to β-TCP bioceramics [42,43]. Copper-based biomaterials show good antibacterial properties and low infection rates after implantation but can potentially lead to toxic reactions [44]. Therefore, the biotoxicity of copper-based biomaterials should be carefully evaluated before their use in bone tissue engineering [45]. At the same time, optimal biocompatibility must also be achieved while maintaining antibacterial properties. Currently, most studies are evaluating the toxicity of copper-based biomaterials by testing them with bone marrow stem cells, osteoblasts, and other cells and using in vivo experiments in animal models [46]. However, they do not stimulate a true toxic response, so testing this biomaterial requires further clinical studies. Recently, there has been promising research on the use of biomaterials containing silver particles in bone tissue engineering. Silver has antibacterial properties through a number of different mechanisms. First of all, it blocks the bacteria’s ability to produce energy by inhibiting the cellular respiratory chain. In addition, silver molecules lead to the release of potassium and bind DNA and RNA, disrupting the processes of translation and transcription. Previous studies have indicated that silver applied to implant coatings shows good antimicrobial activity, especially against Gram-positive bacteria. Unfortunately, data on the efficacy and pharmacokinetic safety of silver in the production of bone substitutes are still limited and inconsistent. The use of silver in bone tissue engineering appears promising, but further research is needed [47]. The general characteristics of different classes of biomaterials are presented in Table 1.

## 3. Natural Biomaterials

Polymers derived from plant or animal sources are referred to as biopolymers. These biopolymers are renewable, biocompatible, and biodegradable. Several types of biopolymers have been comprehensively studied for biomedical applications. Among the natural polymers are the following: chitosan, collagen, gelatine, silk, alginate, cellulose, and hyaluronic acid. In the last decade, hydrogel materials, which can be made of both natural and synthetic polymers cross-linked by covalent or non-covalent chemical bonds, have begun to play an increasingly important role. Hydrogels exhibit a structure similar to the micromolecular components of our bodies, are biodegradable, and promote the formation of new tissue. They are currently used for bone regeneration, in the treatment of damaged cartilage, and as drug carriers. Collagen, alginate, chitosan, PLA, and PPF copolymers, among others, are used to create hydrogels. Natural biomaterials including collagen, chitosan, sodium alginate silk fibroin, and hyaluronic acid can simulate the natural extracellular matrix of bone and then biodegrade to carbon dioxide and water in vivo. Degradation to non-toxic products occurs by hydrolysis and takes a long time due to the high molecular weight. Natural biomaterials are widely used in the preparation of bone tissue engineering scaffolds due to convenient material sourcing, good plasticity, and good biocompatibility. Collagen is a major component of the skin, bone, tendons, and ligaments and has a high swelling index and low antigenicity, making it an ideal natural material for bone tissue engineering. However, its poor mechanical properties limit its direct use as a bone substitute, so attention has turned to a variety of composite scaffolds containing collagen along with high physical strength. After collagen implantation, such immune reactions can occur that can lead to rejection of the implant. To reduce antigenicity, enzymatic digestion is used and then collagen can be prepared for implantation through the use of appropriate techniques. Freezing and freeze-drying techniques can be used for this purpose. Increasing the tensile strength of collagen by tanning can also be achieved. The final procedure for preparing collagen for implantation is sterilisation.

De-proteinized bovine mineral matrix (Bio-Oss) is naturally de-proteinized from the mineral fraction of bovine spongy and cortical bone, which retains a fine beaded structure and internal pores, providing favorable conditions for osteoblast ingrowth and angiogenesis [48]. Bio-Oss bone contains more carbonate to facilitate autologous osteointegration and achieve the required mechanical strength and stiffness [49,50].

Trials have also been conducted using autologous tooth graft material (AutoBT) and autologous dentin particles. Due to the favorable effects on bone tissue processes and the lack of immune rejection, promising clinical results are expected [51]. However, the mechanism of dentin-induced osteogenesis is unclear, and the preparation is cumbersome and time-consuming, which may limit its widespread use in clinical practice [52].

Porous scaffolds made of biomaterials doped with osteoblasts derived from autologous bone tissue have found great interest in tissue engineering. The in vitro osteogenesis of osteoblastic cells from rabbit bone tissue and mesenchymal stem cells from bone marrow was studied on biomaterials based on gelatine-hydroxyapatite (HG) with the addition of lyophilised amorphous chitosan [53]. Recently, various composite nanofibers of chitosan as a base material have been reported for bone tissue engineering. Jing et al. [54] produced aligned poly(propylene carbonate)/chitosan composite nanofibers; chitosan/PVA nanofibers were prepared using multicarboxylic acids as an environmentally-friendly solvent via electrospinning [55,56]. A nanofibrous chitosan scaffold reinforced with silicium nanoparticles was prepared for bone tissue engineering; the results showed that silicium nanoparticles improved the mechanical properties, biodegradability, and bone-forming ability [57].

Silk is a protein-based biopolymer and is of great interest in the field of biomaterials science due to its biocompatibility and structural properties. Silk has very high tensile and compressive mechanical strength due to its structure [58]. Sofi et al. demonstrated fibroin/chitosan composite silk nanofibers for bone tissue regeneration [59]. Belbéoch and Wittmer reported on the in vitro fabrication of silk nanofibers [60,61]. Unalan et al. used silk fibroin as a coating material in bone tissue engineering [62]. In their work, Bhattacharjee et al. used fibroin containing integrin-binding peptide (Arg-Gly-Asp) sequences to produce composite nanofiber scaffolds [63].

Hydroxyethyl cellulose (HEC) is a cellulose derivative in which an ethyl group replaces one or more of the three hydroxyl groups present in each glucopyranoside. The degree of substitution and their relative distribution strongly affect the properties and behaviour of these polymers [64]. Ao et al. prepared composite scaffolds of cellulose-nanohydroxyapatite nanofibers. Cellulose nanocrystals are largely used as reinforcing materials for nanofibers along with some other polymers [65]. Composite nanofibers based on cellulose and its derivatives are being widely studied for bone tissue engineering applications. Cellulose is mainly blended with other functional synthetics and biopolymers, i.e., PCL, gelatine, PVA, silk fibroin, collagen, and polyurethane [66].

The polymeric materials used in tissue engineering can be divided by their origin into natural and synthetic polymers. The first group, the so-called biopolymers, includes polysaccharides (starch, chitin, and hyaluronic acid derivatives) and proteins (collagen and elastin), as well as various types of fibers with a reinforcing function (for example, natural lignocellulosic fibers). The second category includes aliphatic polyesters (polylactide PLA, polyglycolide PGA and their copolymers, and polycaprolactone PCL), as well as polymers from the organosilicon group, for example, polydimethylsiloxane PDMS, polyhydroxyalkanoates PHA, poly propylene fumarate PPF, and polyhydroxybutyrate PHB [67]. An important feature of biopolymers is their ease of processing and resorption into non-toxic substances, such as carbon dioxide and water. The degradation rate of aliphatic polyesters proceeds in the following order: PGA degrades the fastest, followed by PLA, and PCL degrades the longest. Synthetic polymer biomaterials have been extensively studied for bone regeneration, including the commonly used polylactic acid (PLA), polyglycolic acid (PGA), and polylactic and glycolic acid copolymer (PLGA). Polymethylmethacrylate (PMMA)-based bone cement is the bone cement used in clinical practice due to its rapid setting and better mechanical strength. However, it is known to cause mild damage to surrounding bone tissue and its monomer has proven biological toxicity [68]. In addition, the low biodegradation rate of PMMA in the defect area may negatively affect new bone growth, which is not conducive to bone defect regeneration and repair in future clinical use [69]. PVA-based biomaterials and nanofiber scaffolds are used for reconstructive applications in orthopaedics [70]. PVA is a material that is biologically stable in vivo and has suitable biomechanical properties. Due to its chemical and physical properties, it is widely used in biomedical fields such as contact lenses, artificial heart inserts, and wound dressings [71]. Materials based on polymers and calcium triphosphate and hydroxyapatite are used with varying success as sponge bone substitutes. One of the significant problems associated with such composites is the separation of the polymer from the inorganic fraction in an aqueous environment and therefore also in the presence of body fluids. To avoid this, there has been interest in nanocomposites based on bioresorbable polymers and ceramics in which the crystal sizes do not exceed 200 nanometres. It is suggested that by using crystals of this size, it will be possible to obtain implants with enhanced mechanical properties and avoid the delamination process at the boundary between the organic phase of the polymer and the mineral phase [72]. Song et al. prepared composite scaffolds of PVA/collagen/hydroxyapatite nanofibers and found that nano-HA and collagen interacted with PVA particles to enhance the hydrolytic resistance and mechanical properties of the nanofibers, which provided long-term stability [73]. The combination of nano-HA and collagen also increased the adhesion and proliferation of mouse bone cells (MC3T3) in vitro [74]. Liao et al. and reported that the incorporation of MWCNTs did not significantly affect the morphology of PVA/CS nanofibers; importantly, the ability of nanofibers to adsorb proteins was significantly improved [75].

In vitro cell culture of mouse fibroblasts implanted on electrospun scaffolds showed that the incorporation of MWCNTs into PVA/CS nanofibers significantly promotes cell proliferation [75]. Polyethylene oxide (PEO) is a crystalline, water-soluble thermoplastic polymer. It is also known as polyethylene glycol (PEG) or polyoxyethylene (POE), depending on its molecular weight. POE is widely recommended for medical applications due to its very low toxicity and water solubility. Talebian et al. have produced composite chitosan/POE/bioactive glass nanofibers for bone tissue regeneration, which have shown great potential for future clinical reconstructive applications [76]. Poly(caprolactone) (PCL) is a biocompatible, absorbable, and low-cost synthetic polymer, which is synthesised by the ring-opening polymerisation reaction of ε-caprolactone with a catalytic support, i.e., cinnabar octanoate. Due to its semi-crystalline and hydrophobic nature, it exhibits very slow degradation (2–4 years depending on the initial molecular weight) and has mechanical properties suitable for various applications [77,78]. PCL and electrospun composite nanofibers have been widely studied as potential biomaterials for bone scaffolds. Ren et al. fabricated scaffolds from electrospun nanofibers with a PCL/gelatine core [79]. In recent years, PCL nanofiber scaffolds have been accepted as a potential biomaterial for use in bone tissue due to their ease of design and fabrication, making them suitable for biological applications. PLGA polyester is a copolymer of lactic acid (PLA) and polyglycolic acid (PGA). It is the best-defined biomaterial available for drug delivery in terms of structure and performance. The enantiomeric forms of PLA polymer are poly-D-lactic acid (PDLA) and poly-L-lactic acid (PLLA) [80].

The local delivery of antimicrobials from polyurethane scaffolds has been studied as an effective strategy for preventing infection [81,82]. Tobramycin is an aminoglycoside antibiotic that acts mainly on Gram-negative microorganisms, but particularly on *S. aureus*, which is the microorganism that is most commonly associated with the cause of osteitis [83]. In addition, topical delivery of tobramycin from cemented implants containing PMMA is a widely used clinical therapy for treating infected fractures [83]. An important limitation of PMMA implants is that they must be removed before placement of the final bone graft. Therefore, the use of a biodegradable polyurethane scaffold with drug-releasing properties may prove beneficial in reducing the number of surgical procedures required. The use of injectable polyurethane containing tobramycin encapsulated in PLGA microspheres has demonstrated both space-holding mechanical properties and prolonged release of the antibiotic for up to 2 weeks [84]. The release kinetics from polyurethane scaffolds were comparable to drug release from PMMA and calcium sulphate bone cements and exceeded the minimum inhibitory concentration (MIC, 4–8 μg/mL) as well as the minimum bactericidal concentration (MBC, 16 μg/mL) for tobramycin against *S. aureus* [85]. The addition of PEG to binary polyurethane increased the release kinetics of tobramycin due to the increased hydrophilicity of the polymer [84]. Polylactic acid (PLA) is an aliphatic polyester composed of lactic (2-hydroxypropionic) acid molecules. It is also a biodegradable thermoplastic derived from renewable plant sources such as starch and sugar [86]. PLA has been successfully processed into a fiber form and is mainly used for tissue engineering and biomedical applications [87,88]. Chae et al. fabricated hybrid scaffolds of anhydrous dicalcium phosphate/polylactic acid nanofibers and showed that dicalcium phosphate significantly increased bone cell adhesion, differentiation, and mineralisation on PLA nanofibers [89]. In recent years, due to the rapid development of polymeric materials, polyetheretherketone (PEEK), as a new high-performance biocompatible polymer, has been approved by the FDA as an implantable material and is gradually becoming more widely used in the biomedical field. An overview of the most important biomaterials with a comparison of their physical and chemical properties is presented in Table 2.

## 4. New Research Directions in the Search for Biomaterials for Bone Tissue Engineering 

Tissue engineering is an effective alternative to traditional methods of treating damaged tissues and organs. Commonly known methods, such as transplantation or the use of artificial organs, have proven to be quite problematic, mainly due to the possibility of rejection of the transplant by the body, lack of integration with the recipient’s tissue, or limited time of use. The use of tissue scaffolds made of suitable biomaterials makes it possible to regenerate many pathologically damaged tissues (including bone and cartilage, skin, nerve tissue, and blood vessels). Over the past decade, significant progress has been made in the search for new ideal biomaterials capable of meeting the ever-increasing demand for bone substitutes in reconstructive surgery. Intensive research is underway on combining composites with metal ions, osteogenesis-stimulating proteins or mesenchymal stem cells, among others. Huge advances have been made in technologies for spatial modeling of material structure, such as 3D printing and electrospinning. Research in recent years has highlighted the role of manufacturing porous biocompatible scaffolds with good mechanical properties using 3D printing. Stem cells and numerous growth factors were also used in this research. Qiao et al. prepared a hydrogel scaffold composed from of sodium tetraborate (Na_2_B_4_O_7_), polyvinyl alcohol (PVA), Ag NPs, and tetraethyl orthosilicate (TEOS). These 3D composite scaffolds showed very good mechanical and biological properties, inhibiting bacterial growth and promoting bone cell differentiation [90]. Kankala et al. produced a porous scaffold for bone regeneration containing nano-hydroxyapatite (n-HA) and poly(lactide-co-glycolide) (PLGA) using 3D printing and a freeze-drying process [91]. This scaffold had very good mechanical properties and enhanced the growth and differentiation of bone cells.

A number of new nanomaterials are currently being developed, obtained using new biotechnologies, such as: molecular self-organization technology, wet chemical precipitation, hydrothermal synthesis, freezing phase separation, and sol–gel synthesis [92]. The use of these numerous modern technological methods makes it possible to obtain new nanocomposites characterized by very good mechanical properties and favorable effects on bone cell proliferation and differentiation.

In recent years, genetic engineering methods have also been used to create novel biomaterials for bone regeneration [93]. Through this research, there is a chance to find genes and regulatory RNAs that modulate the expression of proteins that affect bone formation processes, as well as the bone destruction and immune processes involved in bone regeneration.

Future studies of the properties of various nanocomposites combined with stem cells should improve the results of the reconstructive treatment of bone defects by enhancing osteointegration. Of the several groups of biomaterials used in tissue engineering, biodegradable polymers have the greatest potential for use. A scaffold made of such a material degrades in a specific way, at a time adapted to the rate of cell proliferation. This eliminates the need to remove the implant from the body at the end of the treatment process, which increases the probability of success of the applied therapy. There are still many unanswered questions that may have important implications for the role of nanostructured biomaterials in bone regeneration. Advanced tissue engineering technologies may represent a breakthrough in overcoming the limitations of the current biomaterials used for bone tissue substitution by providing precise control of biochemical and physical properties.

## 5. Conclusions

In conclusion, the combination of appropriately selected biomaterials and tissue engineering techniques will enable efficient and reproducible fabrication of new implants that more ideally mimic the dynamic properties of the microenvironment during the development of regenerating bone tissue. New research directions should be aimed at better mimicking the natural processes of bone regeneration, such as the coupling between angiogenesis and osteogenesis, which may require the recruitment and differentiation of progenitor cells. Although it is difficult to mimic nature, recent scientific and technological discoveries have shown the potential to create bone scaffolds that would support local and systemic biological functions. The right choice of scaffold materials, their geometry, pore size, and ability to release biomolecules at the desired rate will play a key role in the future development of bone scaffolds.

## Figures and Tables

**Table 1:** General characteristics of different classes of biomaterials.

	Biopolymers	Metals	Bioceramics
Type of connections	Covalent or van der Walls	Metallic	Ionic/covalent
Chemical stability	Weak	Good	Very good
Electrical conductivity	Very low	High	Very low
Thermal conductivity	Very low	High	Low
Characteristics and advantages	Degradable, similar density to soft tissue, and easy to process	High hardness and strength	Non-conductive and biologically inert; optimally imitate the properties of bone
Mechanical strength	Very high strength and plasticity (easy to shape and process)	Resistance to stretching	Brittle and fragile
Main disadvantages	Thermally unstable and low strength	Wear and corrosion	High density and brittleness
Biomedical applications	Soft tissue implants, drug delivery systems, and tissue engineering	Orthopaedic and dental implants	Tissue engineering

**Table 2:** Comparison of physicochemical properties of selected biomaterials used in bone tissue substitution.

	Yield Strength [MPa]	Compressive Strength [MPa]	Tensile Strength [MPa]	Young’s Modulus [GPa]	Flexural Strength [MPa]	Density [g/cm^3^]
Cancellous bone		2–20		01–2	15.8	1.0–1.4
Cortical bone	148	100–200	50–151	5–20	≥160	1.80–2.10
Alloy TiAl_6_Nb_7_	≥800	1074–1086	≥900	≥105	895–905	4.51
Alloy Ti_6_Al_4_V	≥795	450–1850	≥860	≥100		4.05
Polyethylene	≥21		38–49	≥3	1.6–1.8	0.942–0.965
Mg Alloys		65–100	170–270	41–45		1.45–2.0
Aluminum oxide ceramic Al_2_O_3_		150	≥500	≥380	400	3.94
Alloy Mg-Zn			67–169.5	42.3–45.3		1.7–2.0
Hydroxyapatite HaP		500–1000	40–300	80–120	38–250	3.10
Porous hydroxyapatite 82–86%		120–900	3	0.83–1.6 × 10^−3^	2–11	
Hydroxycellulose hydrogel		32	3.5	2.23		
Bioglass		800–1200	42	40–140		1.8–29
PEEK	≥90	80	≥100	3–4	≥150	1.3
PLA	4–25		50–70	2.4		1.25
PLGA		2.90–4.19	41.4–55.2	1.4–2.08		
PCL			20.7–34.5	0.21–0.35		1.11
Chitosan fibers			9.0–16.0	250–380		
PLA/chitosan		47.1	44.5		156.9	
Silk			500–740	5–17		

## Data Availability

Not applicable.
